# Lisaftoclax combined with ixazomib and dexamethasone after CAR-T for maintenance therapy in transplant-ineligible relapsed/refractory ultra-high-risk multiple myeloma: two case reports and literature review

**DOI:** 10.3389/fonc.2026.1784393

**Published:** 2026-04-28

**Authors:** Weige Xu, Xue Qiao, Linlin Zhang, Lina Xing, Jingnan Zhang, Xiaonan Guo, Shukai Qiao

**Affiliations:** 1 Department of Hematology, The Second Hospital of Hebei Medical University, Shijiazhuang, Hebei, China; 2 Department of Hematology, The Second Affiliated Hospital of Xingtai Medical College, Xing Tai, Hebei, China

**Keywords:** CAR-T, dexamethasone, ixazomib, lisaftoclax, maintenance therapy, minimal residual disease, multiple myeloma, ultra-high-risk

## Abstract

Multiple myeloma (MM) is an incurable malignancy. The treatment mainly includes induction therapy, minimal residual disease (MRD) clearance therapy, and maintenance therapy. For high-risk/ultra-high-risk (UHR) or relapsed/refractory MM, optimizing the eradication of MRD and sustaining long-term suppression of MRD at low levels are a formidable challenge. Ixazomib (I) is a reversible proteasome inhibitor (PI) that is available orally as the prodrug ixazomib citrate. Lisaftoclax is a novel, potent, selective BCL-2 inhibitor under clinical development for the treatment of patients with hematologic malignancies or solid tumors and has shown clinical antitumor benefit. Herein, we report two patients with relapsed/refractory UHR MM who achieved durable disease control with MRD negativity after receiving ixazomib, lisaftoclax, and dexamethasone (ILD) as maintenance therapy following B Cell Maturity Antigen BCMA–chimeric antigen receptor (CAR)-T cell therapy. Regarding the treatment regimen, all drugs were administered orally: ixazomib 4 mg d1, d8, and d15; lisaftoclax 400 mg d1–d14; and dexamethasone 20 mg d1, d8, and d15. One treatment cycle is defined as 28 days. Treatment will be discontinued in the event of uncontrollable active infection or organ injury. These cases demonstrate that the ILD combination therapy can optimize MRD eradication and sustain long-term MRD suppression, offering a promising therapeutic option for patients with limited treatment choices. Formal evaluations of this regimen in patients with high-risk/ultra-high-risk or relapsed/refractory MM may be meaningful. This study was supported by the China Cancer Foundation (Project No.: CFC2023WJZD003).

## Introduction

Patients with relapsed/refractory multiple myeloma (RRMM) have a dismal prognosis. After multiple lines of treatment, they have limited therapeutic options and an extremely short overall survival. To date, no standard maintenance treatment regimen has been established for patients following chimeric antigen receptor T-cell (CAR-T) therapy. Proteasome inhibitors (PIs), immunomodulatory imide drugs (IMiDs), and glucocorticoids like dexamethasone and daratumumab are classic therapeutic drugs. Most patients receive multidrug combination therapy or treatment based on new small-molecule inhibitors because they have at least three lines of drug resistance before receiving the first CAR-T treatment; however, the response rates were low (1).

Ixazomib (I) is a reversible, boron-containing PI that is available orally as the prodrug ixazomib citrate. It particularly inhibits the chymotrypsin-like activity of the β5 subunit of the 20S proteasome. Following its market approval, ixazomib has been extensively utilized in the management of multiple myeloma, particularly during the subsequent maintenance therapy phase. Some results suggest potentially high efficacy and a good safety profile of IRd therapy in patients with RRMM and unfavorable cytogenetics (2).

Lisaftoclax (L) is a novel, potent, selective BCL-2 inhibitor under clinical development for the treatment of patients with hematologic malignancies and has shown clinical antitumor benefit (3, 4). Compared with traditional BCL-2 inhibitors (e.g., venetoclax), lisaftoclax has higher binding affinity to the BCL-2 protein and lower off-target effects, making it more suitable for long-term oral maintenance therapy. Thus, we assessed this three-drug combination therapy in this study.

According to the mSMART 3.0 risk stratification system and the 2024 Revised Guidelines for the Diagnosis and Treatment of Multiple Myeloma in China, ultra-high-risk (UHR) multiple myeloma (MM) is defined as the presence of ≥2 high-risk cytogenetic abnormalities (HRCAs), TP53 biallelic deletion/mutation, primary plasma cell leukemia, or early treatment failure (progression/relapse within 18 months after intensive therapy). HRCA includes del(17p)/TP53 mutation, t(4;14), t(14;16), t(14;20), and 1q21 amplification (≥4 copies). Patients with UHR MM have a worse prognosis and are insensitive to conventional therapy, with unmet clinical needs for effective maintenance therapy after CAR-T treatment.

Herein, we report two patients with relapsed/refractory UHR MM who achieved durable disease control and sustained MRD suppression with the ILD maintenance regimen after CAR-T cell therapy. The regimen was safe and effective and may provide a new option for relapsed or refractory MM patients with high-risk/ultra-high-risk characteristics.

## Methods

### Efficacy evaluation

Efficacy assessment was performed in accordance with the 2026 International Myeloma Working Group (IMWG) Response Criteria for Multiple Myeloma, including complete response (CR), very good partial response (VGPR), partial response (PR), stable disease (SD), and progressive disease (PD). Minimal residual disease (MRD) was assessed by 8-color multiparameter flow cytometry (MFC; sensitivity 10^−5^) for bone marrow samples and protein immobilization electrophoresis for blood and urine specimens. Whole-body ^18^F-FDG PET/CT was used for imaging-based MRD evaluation, with no abnormal metabolic foci defined as imaging MRD negativity. Consecutive MRD negativity was defined as double negativity of MFC, M protein, and PET/CT at two consecutive time points with an interval of ≥1 month.

### Safety evaluation

Adverse events (AEs) were graded according to the Common Terminology Criteria for Adverse Events (CTCAE) v5.0. The duration, management, and outcomes of AEs were recorded in detail. Dose reductions or interruptions of the ILD regimen due to AEs were also documented.

## Case presentations

### Patient 1

A 61-year-old female patient was diagnosed with MM 3 years ago at our department. Initial PET/CT revealed extramedullary involvement (EMI) manifesting as hypermetabolic soft tissue nodules in the left pedicle of the third cervical vertebra and the space between the left psoas major muscle and the second lumbar vertebra. Bone marrow aspiration showed 8.0% myeloblasts, 8.0% immature plasma cells, and 47.5% mature plasma cells, with 1.0% myeloblasts, 2.0% immature plasma cells, and 15% mature plasma cells detected in peripheral blood. Flow cytometry identified 54.21% abnormal plasma cells in bone marrow nucleated cells.

Fluorescence In Situ Hybridization (FISH) analysis (MM-sorted cells) confirmed CDKN2C deletion (85%), RB-1 deletion (70%, suggesting −13), and IGH–MAF fusion gene positivity (90%). Karyotype analysis revealed complex abnormalities: 45, X, del(1)(p13), +3, add(3)(q22), add(5)(p15), add(7)(p15), del(9)(q12q31), der(11)t(4,11)(q24q23), −13, del(13)(q14q33), −16[3]/46, XX [7]. This patient harbored ≥2 high-risk cytogenetic abnormalities, meeting the UHR MM criteria. Serum immunofixation electrophoresis detected a monoclonal IgG-κ component in the γ region, with M protein accounting for 57.8% (72 g/L) and a free light chain (FLC) κ/λ ratio of 164.5978. The patient initially received Dara + VRd chemotherapy. Eight courses later, evaluation in May 2023 demonstrated a CR. Subsequent lenalidomide maintenance therapy was administered.

In February 2024, the patient experienced her first relapse. Plasma cells and M protein were slightly increased. Fluorescence In Situ Hybridization (FISH) confirmed CDKN2C, TP53, and RB-1 deletions, as well as IGH–MAF fusion. PET/CT showed a new soft tissue lesion in the second lumbar paravertebral region. Nine courses of XKPD regimens were administered sequentially, achieving a VGPR.

In December 2024, a second relapse occurred with disease progression: bone marrow aspiration showed 1.0% myeloblasts, 5.0% immature plasma cells, and 4.0% mature plasma cells; flow cytometry detected 7.06% monoclonal plasma cells, serum IgG-κ M protein (12.8 g/L, 19.3%), and an FLC κ/λ ratio of 32.1123. PET/CT showed diffuse bone metabolic hyperactivity, with newly identified abnormal metabolic foci (EMI) involving the right third rib, left fifth posterior rib, sternal manubrium, cervical lymph nodes, clavicular region, and mediastinum.

B Cell Maturity Antigen BCMA-CAR-T cell therapy was initiated on 15 January 2025. MRD assessment on day 28 demonstrated VGPR with M protein negativity and MFC negativity, and PET/CT showed reduced metabolic activity of lesions with no new abnormal foci. At day 65 after CAR-T, serum IgG-κ M protein was 1.5 g/L (2.5%) and the FLC κ/λ ratio was undetectable, maintaining VGPR; MRD assessment showed MFC MRD negativity, and PET/CT showed no abnormal metabolic foci (imaging MRD negativity).

The patient initiated ILD maintenance therapy on day 70 after CAR-T infusion, with no biochemical or radiographic progression before therapy initiation. Regarding the treatment regimen, all drugs were administered orally: ixazomib 4 mg d1, d8, and d15; lisaftoclax 400 mg d1–d14; and dexamethasone 20 mg d1, d8, and d15. The patient achieved CR again at 3 months after ILD initiation, with consecutive MRD negativity (MFC and M protein negativity) confirmed at two consecutive time points (month 3 and month 6 after ILD), and has been receiving outpatient treatment and follow-up to date. As of January 2026, the patient had a recurrence-free survival for 12 months.

During the ILD treatment period, adverse events were graded according to CTCAE v5.0: grade 1–2 thrombocytopenia (lasting for 2 weeks) and localized herpes zoster. Thrombocytopenia was managed with symptomatic supportive treatment (recombinant human thrombopoietin) without platelet transfusion; herpes zoster was cured with acyclovir antiviral therapy. The patient received antiviral prophylaxis (valacyclovir) and antibacterial prophylaxis (trimethoprim–sulfamethoxazole) during ILD maintenance therapy, with no dose reduction or interruption of the ILD regimen required. Weekly peripheral blood cell count detection and monthly T-cell subpopulation analysis were performed for immunosuppression monitoring, with no severe infectious complications occurring.

### Patient 2

A 74-year-old male patient was diagnosed with MM IgG-κ type 7 years and 1 month ago at our hospital. According to staging systems, he was classified as Durie -Salmon (D-S) stage IIA, International Staging System (ISS) stage II, and Revised International Staging System (R-ISS) stage II when he was newly diagnosed. Initial workup results were as follows: bone marrow aspiration showed 21% plasma cells. Flow cytometry identified approximately 4% abnormal plasma cells.

Fluorescence In Situ Hybridization (FISH) analysis confirmed trisomy of the CKS1B locus at 1q21.2 (1q21 amplification, a high-risk cytogenetic abnormality), with normal karyotype. Serum immunofixation electrophoresis detected IgG-κ M protein (33.9%, 30.84 g/L) with total protein 90.96 g/L and IgG 4,160 mg/dL. Urine Bence–Jones protein was positive. Serum FLC analysis showed κ > 4,850 mg/L, λ 7.95 mg/L, and κ/λ ratio >610.0629; urinary FLC revealed κ > 4,850 mg/L, λ 7.72 mg/L, and κ/λ ratio >628.2383. After three courses of Bortezomib, Cyclophosphamide, and Dexamethasone regimen (BCD) and three courses of Bortezomib and Dexamethasone regimen (BD), efficacy assessment was PR on June 26, 2019. Meanwhile, a small number of cells with t(14;16)(q32;q23) (below the probe threshold, a typical HRCA) were detected. Four courses of Bortezomib, Lenalidomide, and Dexamethasone regimen (BRD) later, reassessment at the 11th hospitalization (December 23, 2019) achieved VGPR. The patient continued the Bortezomib, Lenalidomide, and Dexamethasone regimen (BRD) regimen for a total of 10 courses. The M protein slightly decreased but remained persistently positive.

Disease progression (PD) was confirmed at the 22nd hospitalization (April 1, 2022): bone marrow aspiration showed 8.5% plasma cells, elevated M protein in serum and urine immunofixation electrophoresis, and lumbar compression fracture on CT with concurrent waist pain. Karyotype revealed complex chromosomal abnormalities: 56,XY, +5, add(5)(p10), +7, del(7)(q21q31), add(8)(q23), +9, add(10)(p14), +11,+12, add(12)(q24), +15,+15, +18,+mar1, mar2[13]/46,XY [28]. The patient received two courses of the DRd (daratumumab + lenalidomide + dexamethasone) regimen, followed by eight courses of the DPD regimen after pomalidomide approval, maintaining VGPR.

Second disease progression occurred on December 18, 2023, manifested as a significant increase in M protein. Subsequent salvage therapies, including Dara and selinexor, failed to achieve therapeutic efficacy. This patient developed 1q21 amplification, t(14;16), and complex chromosomal abnormalities during disease progression (≥2 HRCA), accompanied by multiple relapses, meeting the UHR MM criteria. B Cell Maturity Antigen BCMA-CAR-T cell therapy was administered on April 16, 2025. MRD assessment at day 28 post-CAR-T cell infusion achieved PR with MFC MRD negativity and IgG-κ M protein positivity (5.5%, 3.3 g/L); PET/CT showed stable disease with no new lesions.

The patient initiated ILD maintenance therapy on day 200 after CAR-T infusion, with persistent low-level M protein expression and no biochemical or radiographic progression before therapy initiation. Since November 2025, the patient has received outpatient maintenance therapy with ixazomib, lisaftoclax, and dexamethasone, maintaining sustained VGPR to date.

During the ILD treatment period, adverse events were graded according to CTCAE v5.0: grade 1–2 nausea and cytopenia (neutropenia + thrombocytopenia, lasting for 3 weeks). Nausea was relieved with antiemetic drugs, and cytopenia was managed with granulocyte colony-stimulating factor (G-CSF) and recombinant human thrombopoietin. No dose reduction or interruption of the ILD regimen was required. The patient received routine infection prophylaxis and immunosuppression monitoring (weekly blood cell count and monthly immune function assessment), with no severe AEs or infectious complications occurring.

## Discussion

Case 1 involves a patient with multiple ultra-high-risk characteristics, including a complex karyotype, ≥2 high-risk cytogenetic abnormalities (CDKN2C deletion, RB-1 deletion, TP53 deletion, IGH–MAF fusion), recurrent EMI, and multiple lines of prior therapy, fully meeting the UHR MM criteria defined by the mSMART 3.0 and 2024 Chinese MM Guidelines. Case 2 involves a patient under long-term follow-up who presented with 1q21 amplification at initial diagnosis and developed t(14;16) during disease progression, with ≥2 HRCA and multiple relapses, also conforming to the UHR MM definition. At initial diagnosis, the latter presented with few adverse prognostic factors and demonstrated sensitivity to the VRd triple-drug combination regimen; however, the disease progressed recurrently with the emergence of a complex chromosomal karyotype, resulting in failure to reduce tumor burden. Literature on analogous cases has shown that even following auto-hematopoietic stem cell transplantation (HSCT) or CAR-T therapy, early relapse may still occur (5).

In our management, we administered ILD maintenance therapy subsequent to CAR-T treatment, with an interval of 70 and 200 days from CAR-T infusion to ILD initiation for the two patients. MRD has remained at low levels to date. Notably, the entire ILD treatment course for both patients was conducted on an outpatient basis, featuring convenient oral administration and favorable safety profiles.

MM is an incurable malignancy. The treatment mainly includes induction therapy, MRD clearance therapy, and maintenance therapy. Over a decade ago, the primary therapeutic modalities for MM were limited to chemotherapy and HSCT, and patient survival outcomes remained dismal (6, 7). The advent of PIs and IMiDs has since revolutionized the management of MM, substantially improving treatment responses and clinical outcomes. The subsequent introduction of CD38 monoclonal antibodies has further addressed the challenge of drug resistance to PIs and IMiDs, representing a pivotal advance in the therapeutic armamentarium against MM (8). With the sequential combination of multiple therapies, such as DRd/VRd–IRd, an increasing number of patients have achieved “functional cure”, and the treatment options for multiple myeloma are increasingly oriented toward long-term chronic disease management (9).

However, patients with high-risk/ultra-high-risk characteristics or recurrent EMI are still insensitive to conventional therapy or frequently relapse after remission. Chemotherapy based on proteasome inhibitors or immunomodulatory drugs is ineffective in controlling the progression of relapsed/refractory multiple myeloma. Physicians have conducted numerous attempts, including intensive chemotherapy, Dara-based combination therapy, bispecific antibodies, and CAR-T therapy (10–13). CAR-T therapy demonstrates an exceptionally high early response rate, yet both post-response treatment and salvage therapy after relapse remain challenging (10, 14, 15).

Ixazomib (I) is a reversible PI that is available orally as the prodrug ixazomib citrate. It particularly inhibits the chymotrypsin-like activity of the β5 subunit of the 20S proteasome. Following its market approval, ixazomib has been extensively utilized in the management of multiple myeloma, particularly during the subsequent maintenance therapy phase. Some results suggest potentially high efficacy and a good safety profile of IRd therapy in patients with RRMM and unfavorable cytogenetics (2, 16, 17).

BCL-2 inhibitors have demonstrated robust therapeutic efficacy in Acute Myeloid Leukemia (AML), Myelodysplastic Syndromes (MDS), chronic lymphocytic leukemia, and other hematologic malignancies. An increasing number of researchers are focusing on the role of venetoclax-based treatment combinations in relapsed/refractory multiple myeloma (18–20). In a phase III trial (21), “Venetoclax or placebo in combination with bortezomib and dexamethasone in patients with relapsed or refractory multiple myeloma”, 291 patients were randomly assigned to receive venetoclax (n = 194) or placebo (n = 97). With a median follow-up of 18.7 months, the median progression-free survival was 22.4 months (95% CI 15.3–not estimable) with venetoclax versus 11.5 months (9.6–15.0) with placebo [hazard ratio (HR) 0.63 [95% CI 0.44–0.90]; p = 0.010]. This study provides a new approach for the combination therapy of PI, BCL-2 inhibitor, and dexamethasone.

Lisaftoclax is a novel, potent, selective BCL-2 inhibitor under clinical development for the treatment of patients with hematologic malignancies or solid tumors and has shown clinical antitumor benefit (22). In this study, both patients had high BCL-2 protein expression in abnormal plasma cells with no t(11;14) translocation and achieved sustained MRD negativity with lisaftoclax-based combination therapy, providing new clinical evidence for BCL-2 inhibitor therapy in non-t(11;14) UHR MM patients. BCL-2 inhibitor (APG-2575)-induced macrophage repolarization could remodel the tumor immune microenvironment, thus improving tumor immunosuppression and further enhancing antitumor T-cell immunity. Multiplex immunohistochemistry confirmed that patients with better immunotherapeutic efficacy had higher CD86, p-NF-κB p65, and NLRP3 levels, accompanied by lower CD206 expression on macrophages. Collectively, these data provide feasibility for the study on APG-2575 in combination with immunotherapy for tumor treatment (23).

Administration convenience is a critical consideration in the management of MM, particularly for patients with advanced-stage MM. Since MM patients value greater convenience and may prefer oral to injectable medications, all these oral triplet options are of interest to MM patients and physicians. For example, all oral treatments are useful for patients who prefer not to travel to clinics or cannot perform self-injection within the community. In addition, oral treatments are less costly for healthcare providers and require less capacity at treatment centers compared to intravenously administered therapies. These cases demonstrate that the ILD combination therapy can optimize the eradication of MRD and sustain long-term suppression of MRD at low levels, offering a promising therapeutic option for patients with limited treatment choices.

This study has several limitations that should be acknowledged. First, the sample size is extremely small (n = 2), which limits the generalizability of the conclusions. Second, the study is an observational case report with no control group, making it impossible to isolate the independent therapeutic effect of ILD maintenance therapy from CAR-T therapy. Third, the follow-up duration is relatively limited, and long-term efficacy and safety need to be further observed. Fourth, no biomarker-based patient selection was performed, and the predictive factors for the efficacy of the ILD regimen remain to be explored. These findings are hypothesis-generating, and further large-sample, multi-center, prospective controlled clinical studies are needed to verify the efficacy and safety of the ILD maintenance regimen in UHR RRMM patients after CAR-T therapy.

## Conclusion

This study highlights that ILD maintenance therapy can achieve durable disease control in UHR MM patients with complex karyotype, unfavorable genetic mutations, and recurrent EMI after multiple lines of therapy, providing a viable therapeutic option for relapsed/refractory high-risk MM. The ILD regimen features oral administration, a good safety profile, and feasibility for outpatient maintenance, with promising clinical application value. To further validate the aforementioned conclusions, therapeutic observations with large sample sizes and longer-term follow-up are ongoing.

## Data Availability

The original contributions presented in the study are included in the article/Supplementary Material. Further inquiries can be directed to the corresponding author.
